# Supplementary material for: Engineering a multivalent antibody nanoparticle to overcome SARS-CoV-2 Omicron immune evasion
Source: PLoS Pathog. 2025 Dec 8;21(12):e1013744. doi: 10.1371/journal.ppat.1013744 (PMC12697983; doi:10.1371/journal.ppat.1013744)
Supplement: S1 Fig — (A) The binding affinities of 1C4 to the RBDs of spike proteins of SARS-CoV-2 WT, Alpha, Beta, Delta, BA.1, BA.2, BA.2.75, BA.5 and XBB variants were determined by surface plasmon resonance. Colored curves are the experimental traces obtained from surface plasmon resonance (SPR) experiments, and curves indicated the best local fit for the data are used to calculate the KD values by using a 1:1 binding model or steady state affinity. (B) A summary of the KD values of 1C4 calculated from the SPR assays in (A). NA indicates not applicable. (PDF) [file ppat.1013744.s001.pdf]

S1 Fig.

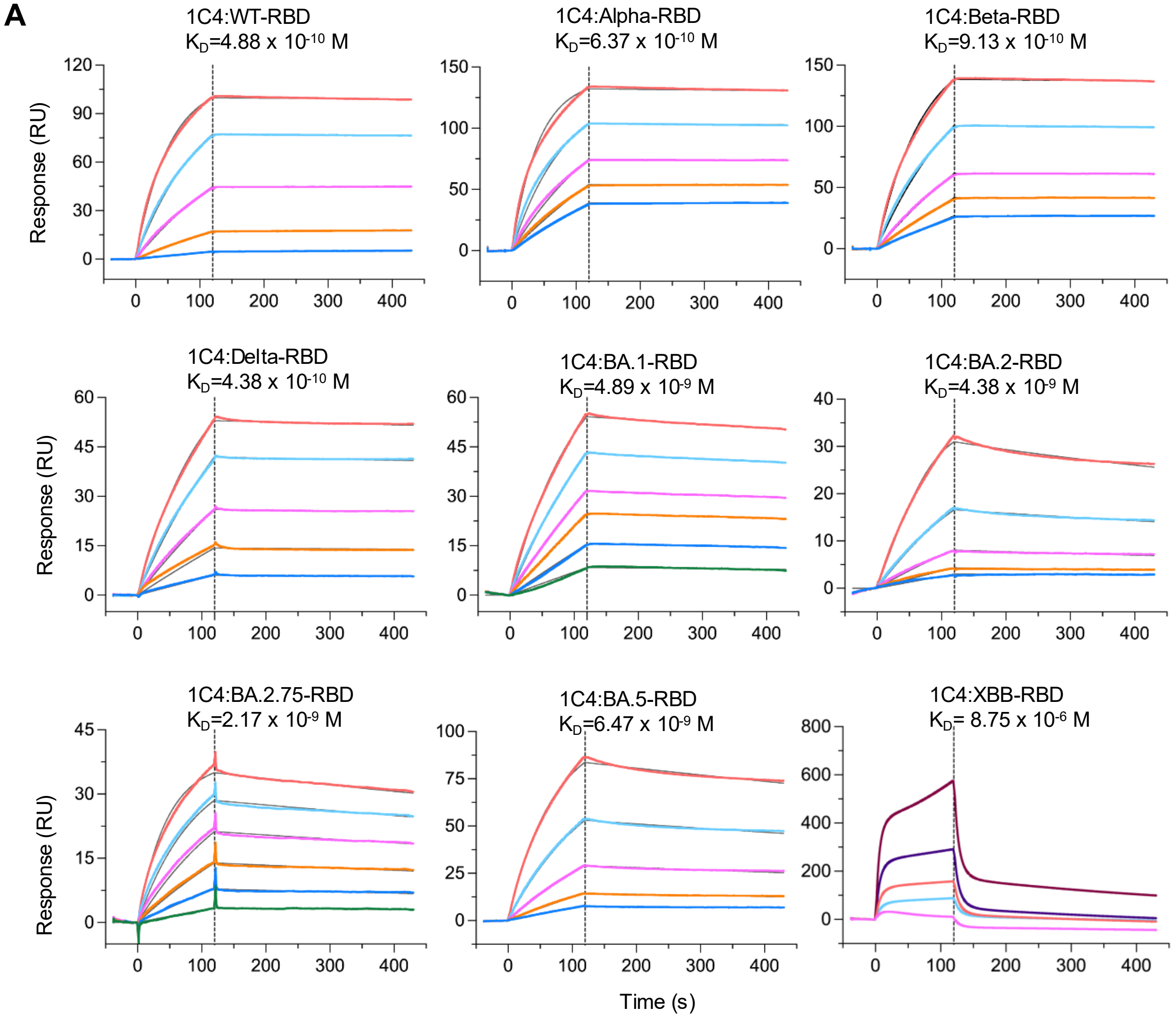

B

Kinetics parameters	mAb 1C4 binding to RBDs of different variants								
	WT	Alpha	Beta	Delta	BA.1	BA.2	BA.2.75	BA.5	XBB
**KD (nM)**	**0.488**	**0.637**	**0.913**	**0.438**	**4.890**	**4.380**	**2.170**	**6.470**	**8,750**
Ka (1/Ms)	4.84 x 10^4	6.04 x 10^4	3.56 x 10^5	1.95 x 10^5	4.81 x 10^4	2.04 x 10^5	2.17 x 10^5	7.04 x 10^4	NA
Kd (1/s)	2.36 x 10^{-5}	3.85 x 10^{-5}	3.25 x 10^{-5}	8.55 x 10^{-5}	2.36 x 10^{-4}	8.91 x 10^{-4}	4.72 x 10^{-4}	4.55 x 10^{-4}	NA
