# Supplementary figures and images for: Engineering a multivalent antibody nanoparticle to overcome SARS-CoV-2 Omicron immune evasion
Source: PLoS Pathog. 2025 Dec 8;21(12):e1013744. doi: 10.1371/journal.ppat.1013744 (PMC12697983; doi:10.1371/journal.ppat.1013744)

S2 Fig.

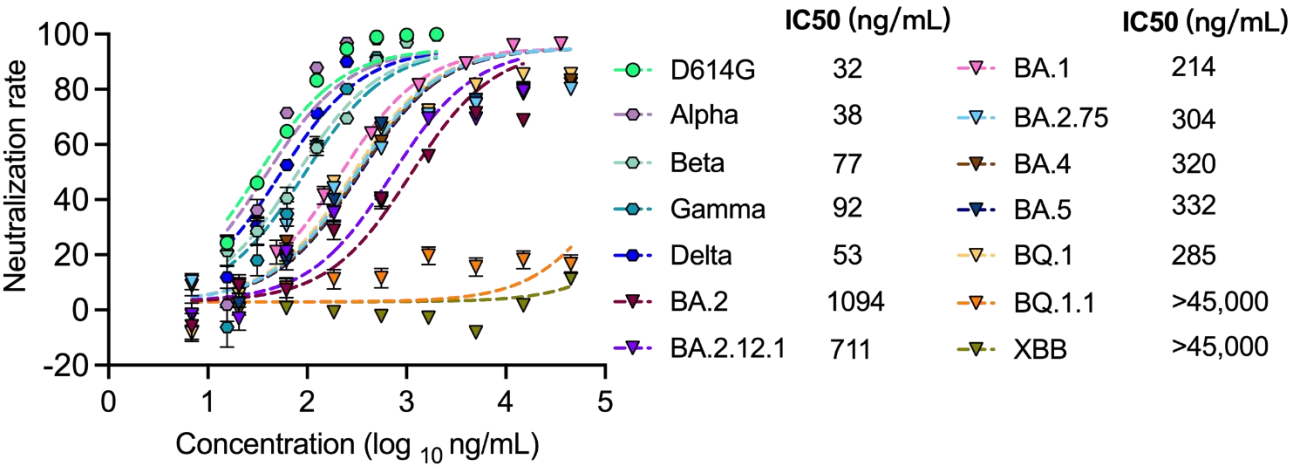

Supplement: S2 Fig — The IC50 values represent the antibody concentration required to achieve 50% neutralization of viral infection. Data were collected from three technical replicates and displayed as means ± SD. (PDF) [file ppat.1013744.s002.pdf]

S3 Fig.

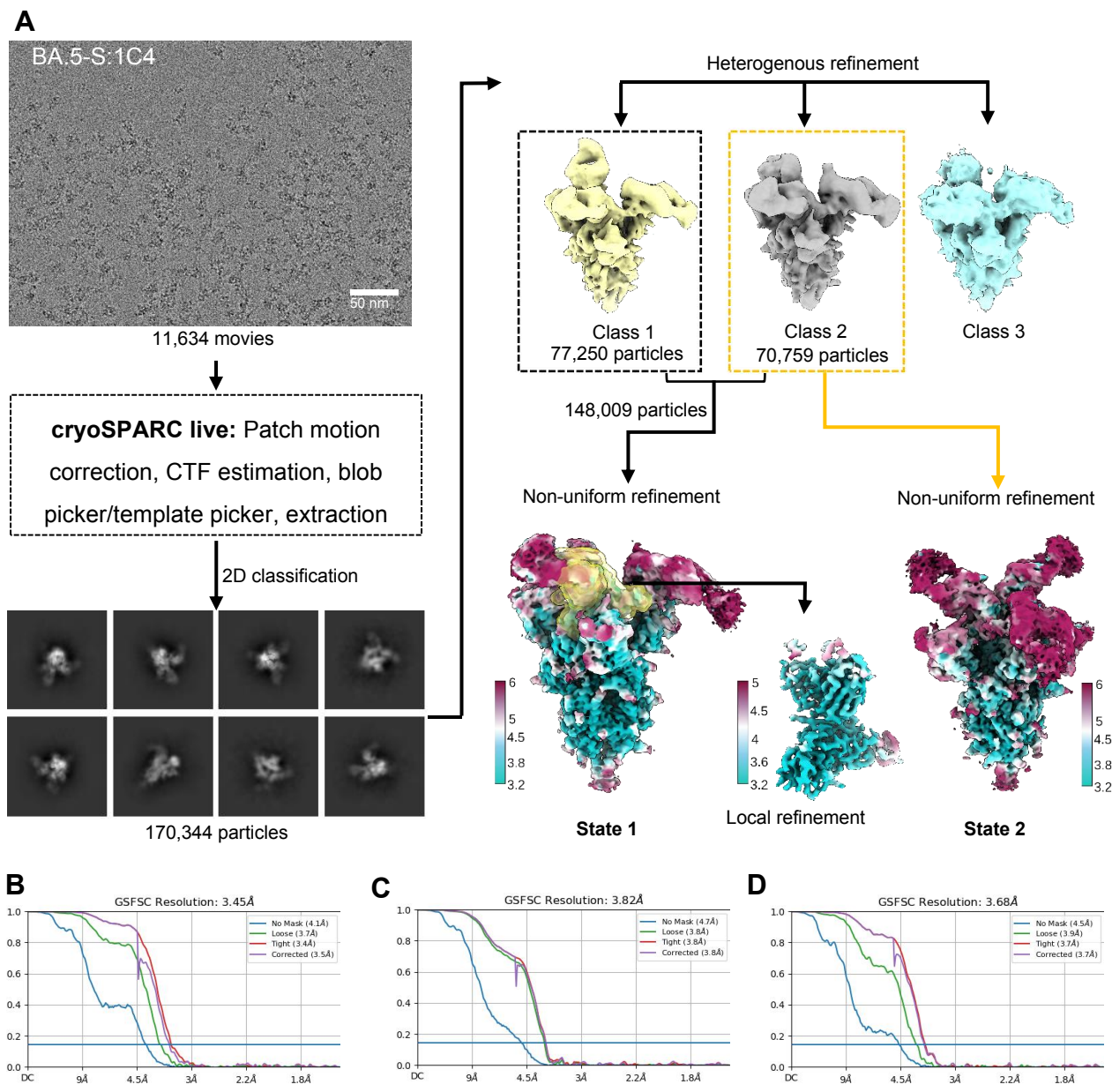

Supplement: S3 Fig — (A) Representative electron micrograph (scale bar: 50 nm), 2D classification results, heterogeneous refinement maps, and final refinement maps (colored by local resolution) are shown. (B-D) FSC curves for the reconstruction of state 1 by global refinement (B) and localized refinement (C), and state 2 by global refinement (D) are shown. (PDF) [file ppat.1013744.s003.pdf]

S4 Fig.

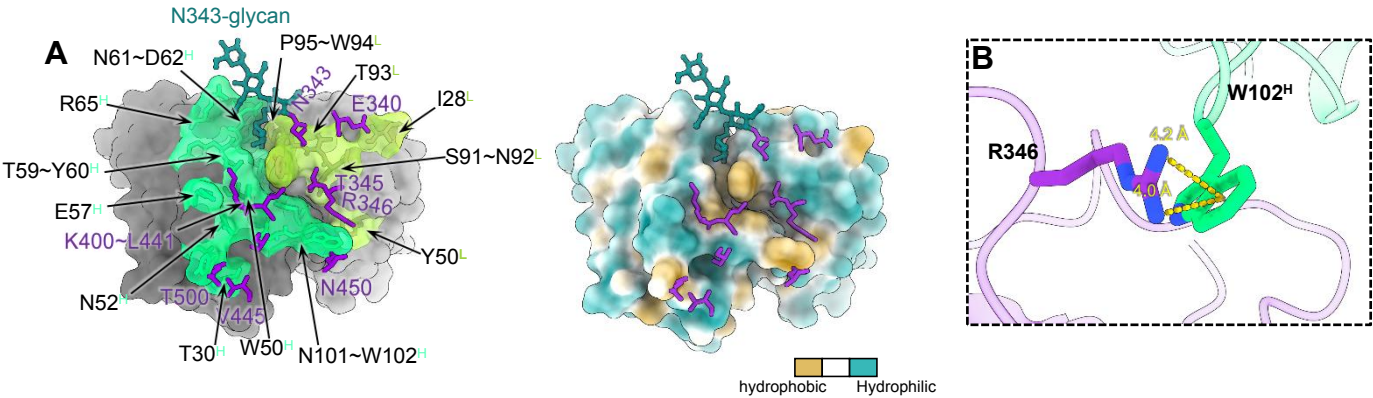

Supplement: S4 Fig — (A) Surface representation of the 1C4 Fab, with interacting residues highlighted as transparent sticks. The corresponding residues on the RBD that form the epitope are shown as purple sticks. Surface hydrophobicity map of the 1C4 paratope.(B) The detail of cation-π interaction between R346 and W102 of 1C4 heavy chain. (PDF) [file ppat.1013744.s004.pdf]

S5 Fig.

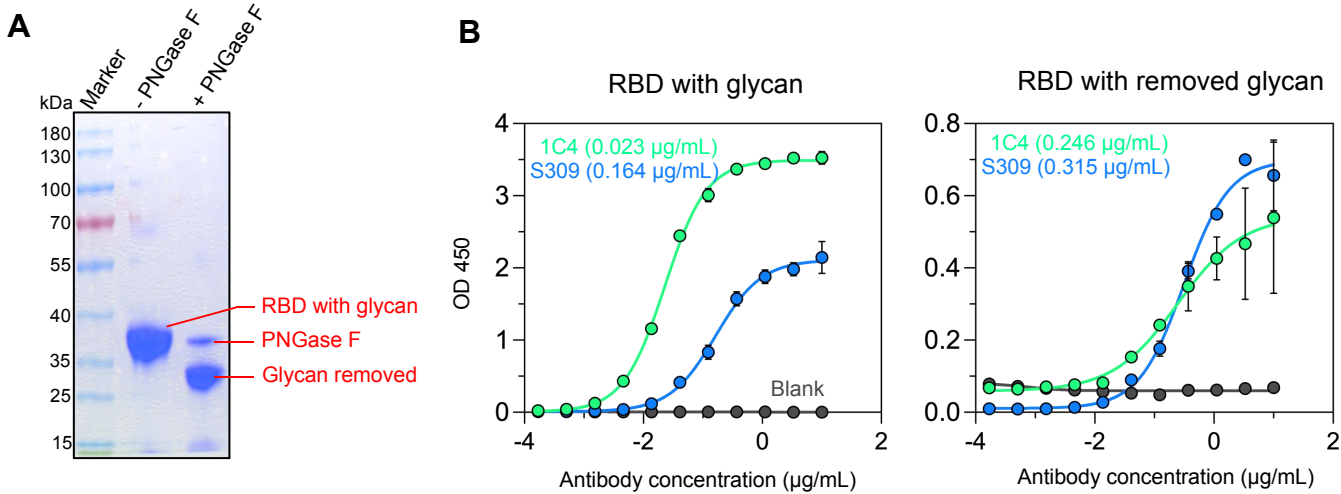

Supplement: S5 Fig — (A) SDS-PAGE analysis illustrates the spike protein RBDs with glycans and after treatment with PNGase F to remove glycans. (B) Impact of glycan removal on 1C4 binding to the RBD. The corresponding 50% effective concentration (EC50) is labeled. (PDF) [file ppat.1013744.s005.pdf]

**S6 Fig.**

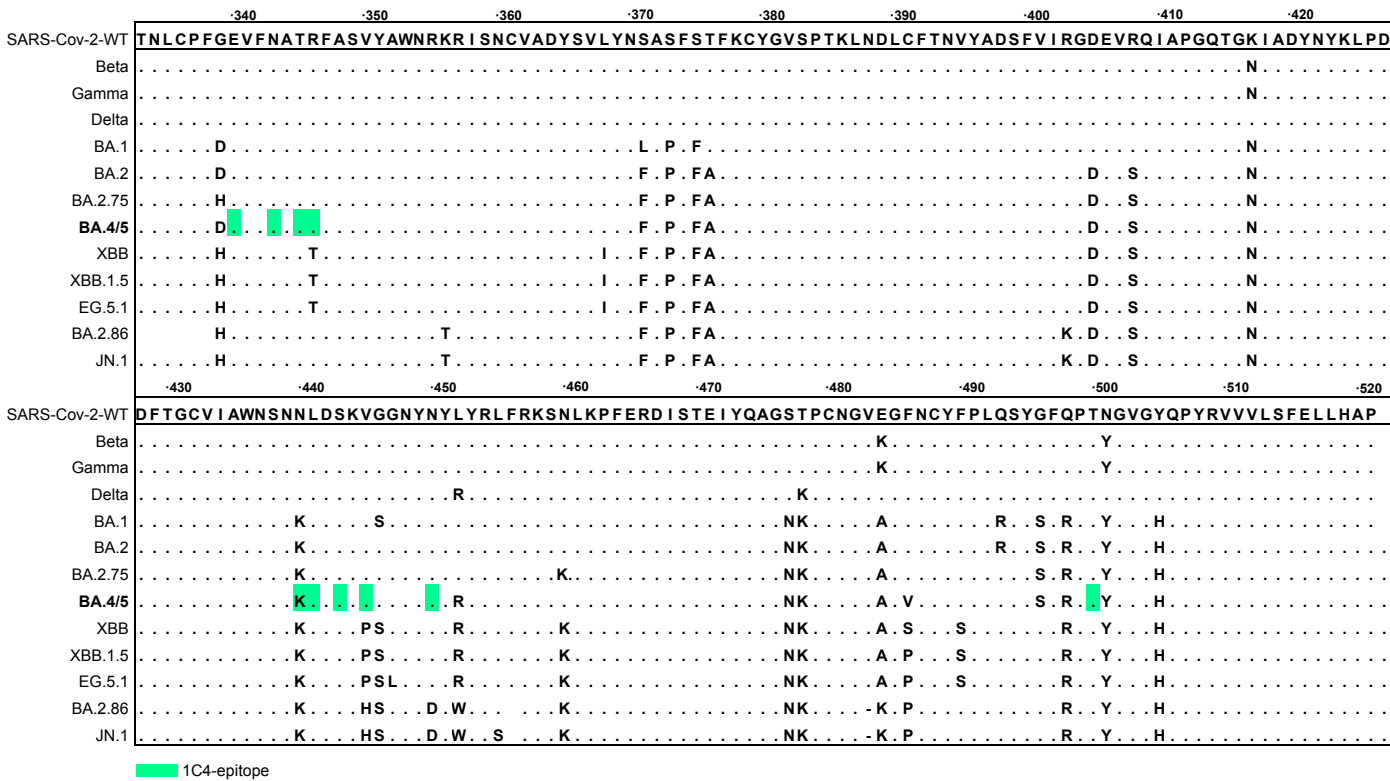

Supplement: S6 Fig — The residues involved in 1C4-footprint highlighted in green. (PDF) [file ppat.1013744.s006.pdf]

**S7 Fig.**

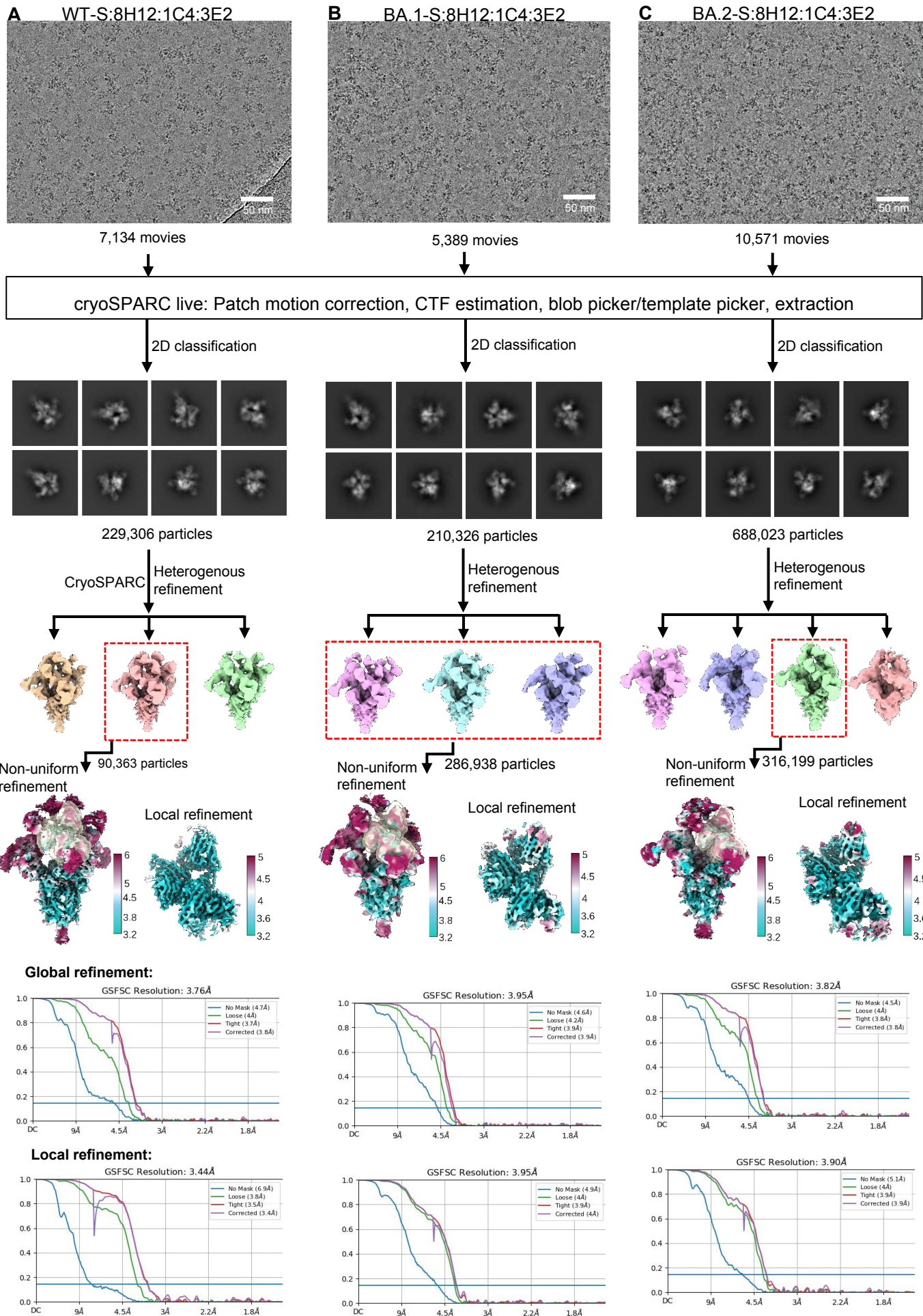

Supplement: S7 Fig — (A-C) Representative electron micrograph (scale bar: 50 nm), 2D classification results, heterogeneous refinement maps, and final refinement maps (colored by local resolution) of WT-S:8H12:3E2:1C4 (A), BA.1-S:8H12:3E2:1C4 (B) and BA.2-S:8H12:3E2:1C4 (C) respectively, are shown. The representative FSC curves for the global reconstruction and localized refinement are also shown. (PDF) [file ppat.1013744.s007.pdf]

S8 Fig.

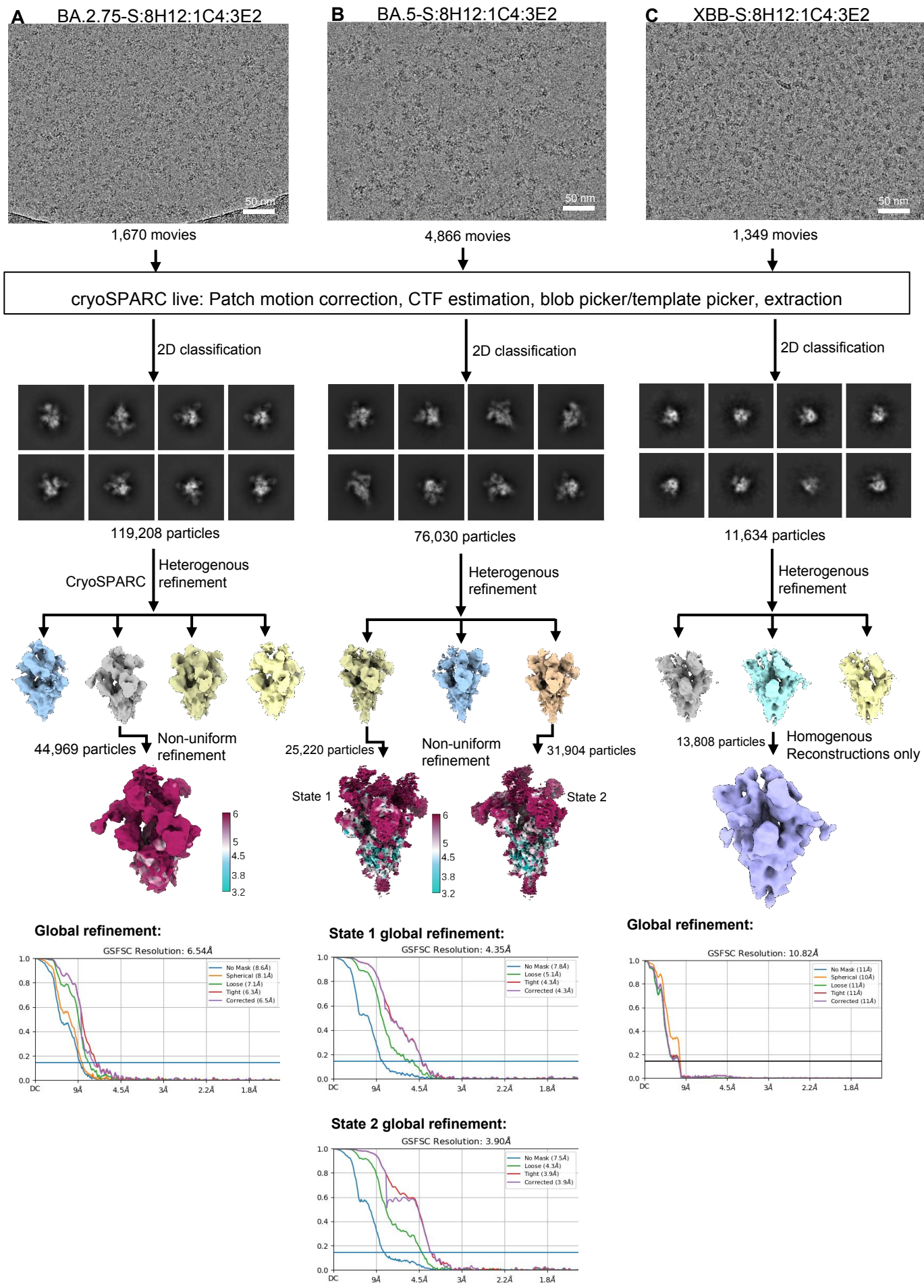

Supplement: S8 Fig — 2.75-S:8H12:3E2:1C4, BA.5:8H12:3E2:1C4 and XBB:8H12:3E2:1C4. (A-C) Representative electron micrograph (scale bar: 50 nm), 2D classification results, heterogeneous refinement maps, and final refinement maps (colored by local resolution) for BA.2.75-S:8H12:3E2:1C4 (A), BA.5:8H12:3E2:1C4 (B) and XBB:8H12:3E2:1C4 (C), respectively, are shown. the representative FSC curves for the global reconstruction and localized refinement are also shown. (PDF) [file ppat.1013744.s008.pdf]

S9 Fig.

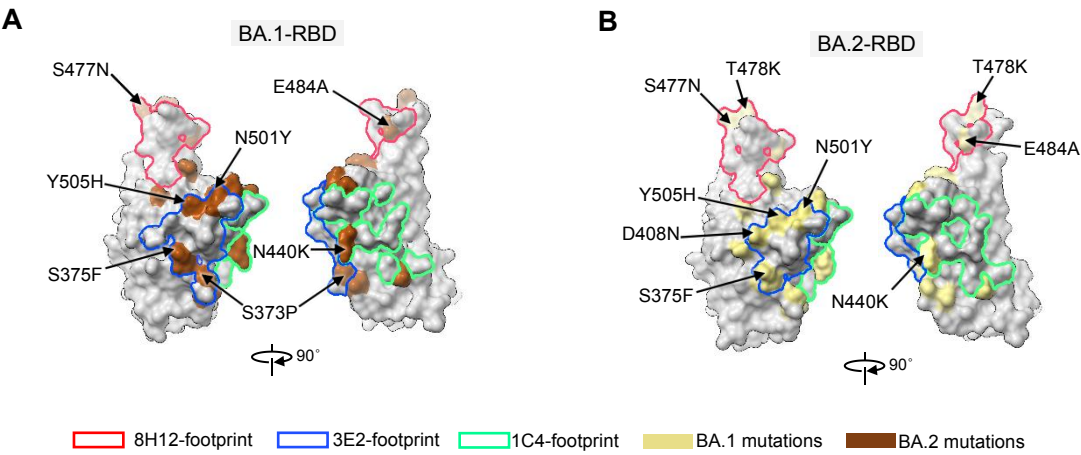

Supplement: S9 Fig — (A and B) The footprints of 8H12, 3E2 and 1C4 are colored by red, blue and green, respectively, on the surface of the BA.1 RBD (A) and BA.2 RBD (B). The BA.1 and BA.2 mutations are highlighted and the residues involved in epitopes of antibodies are labeled. (PDF) [file ppat.1013744.s009.pdf]

S10 Fig.

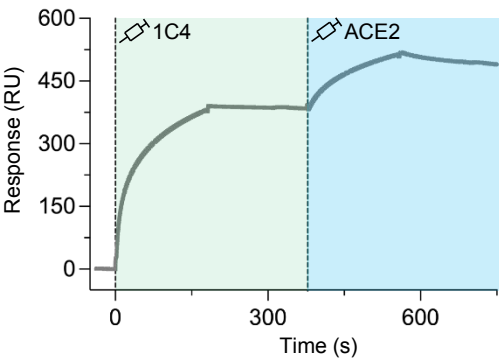

Supplement: S10 Fig — (PDF) [file ppat.1013744.s010.pdf]

**S11 Fig.**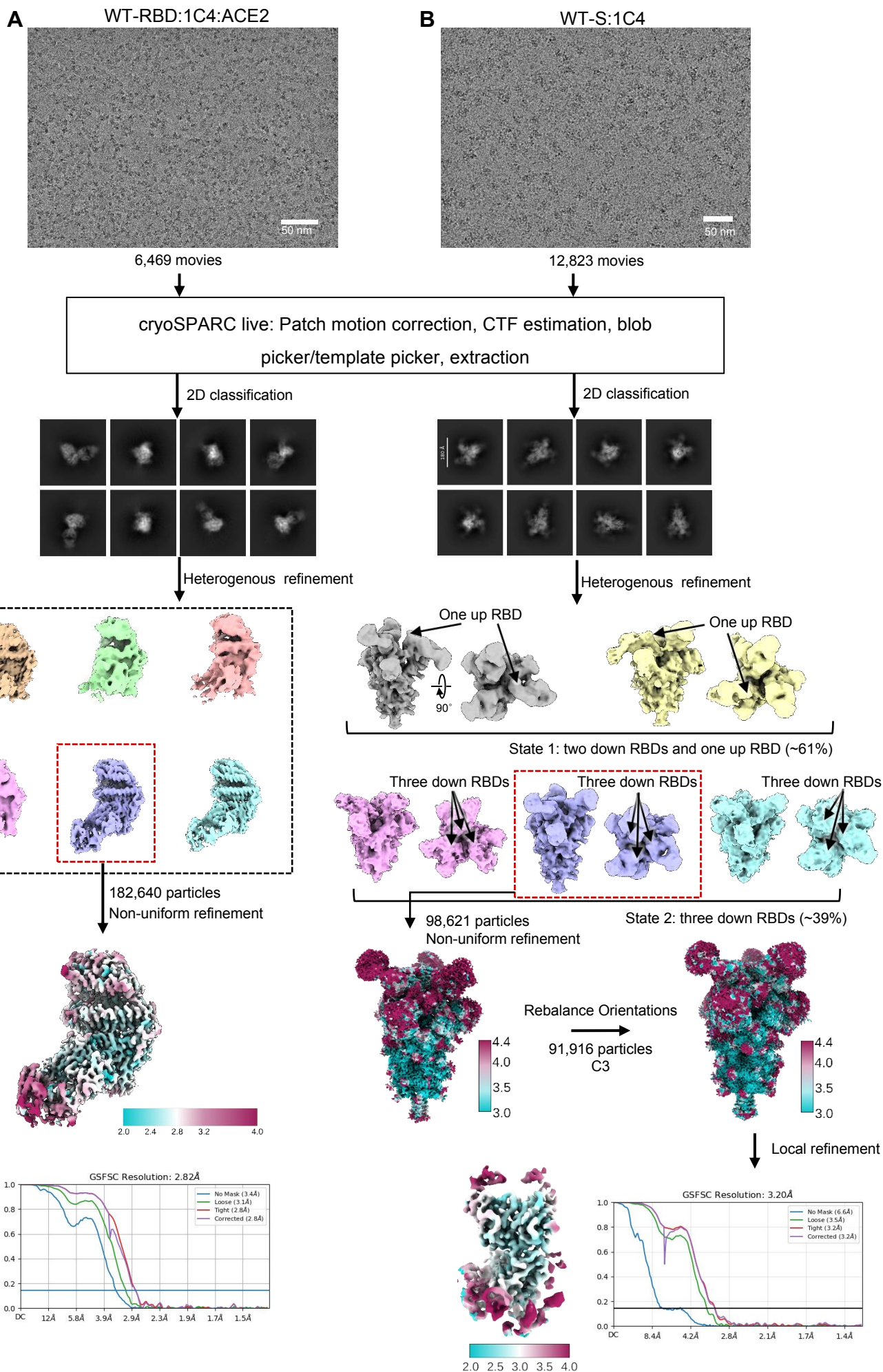

Supplement: S11 Fig — Related to Fig 3. (A and B) Representative electron micrograph (scale bar: 50 nm), 2D classification results, heterogeneous refinement maps, and final refinement maps (colored by local resolution) are shown. (C and D) FSC curves for the reconstruction are shown. (PDF) [file ppat.1013744.s011.pdf]

S12 Fig.

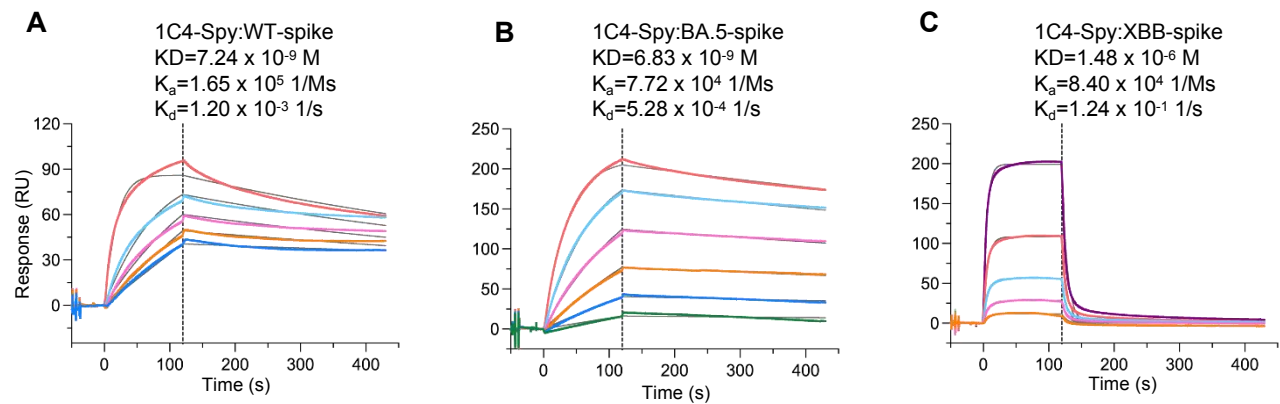

Supplement: S12 Fig — Related to Fig 4. Colored curves are the experimental traces obtained from SPR experiments, and curves indicated the best local fit for the data are used to calculate the KD values by using a 1:1 binding model or steady state affinity. (PDF) [file ppat.1013744.s012.pdf]

S13 Fig.

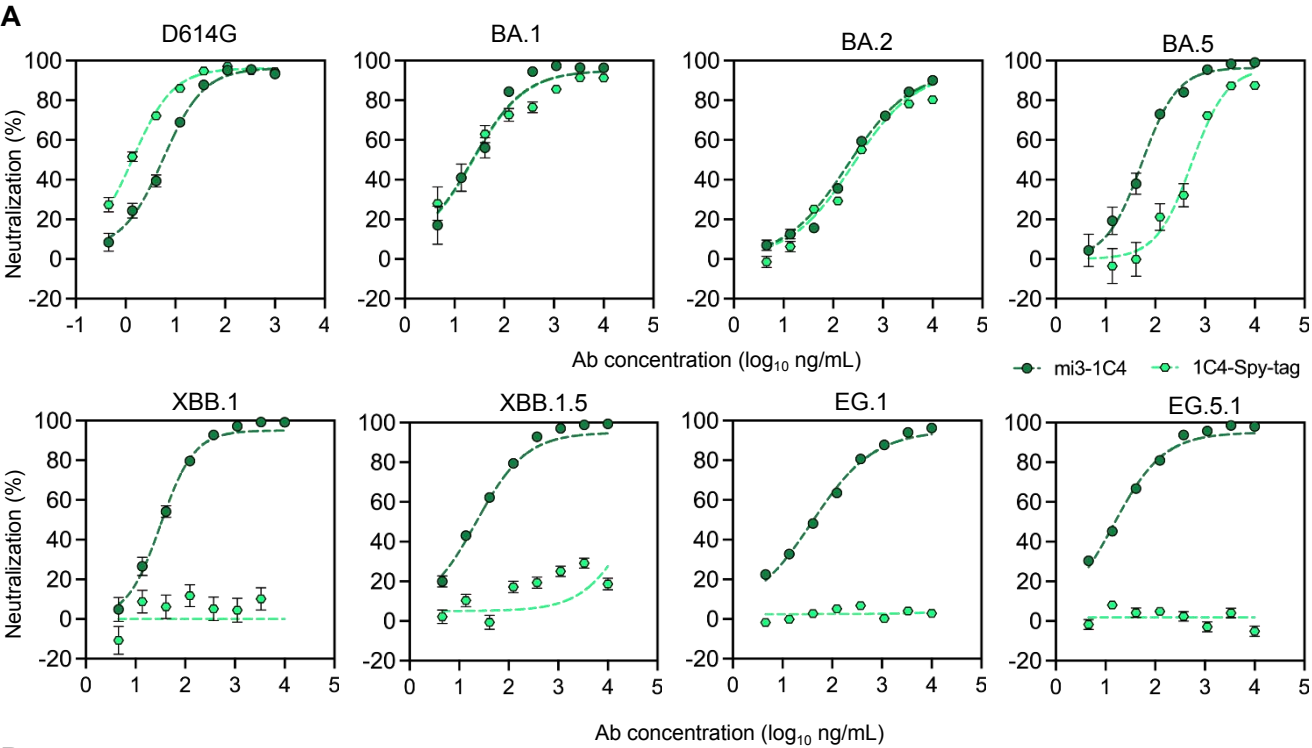

**B**

|             | IC50 (ng/mL) |      |      |      |         |         |         |         |
|-------------|--------------|------|------|------|---------|---------|---------|---------|
| Ab version  | D614G        | BA.1 | BA.2 | BA.5 | XBB.1   | XBB.1.5 | EG.1.   | EG.5.1  |
| mi3-1C4     | 6            | 23   | 264  | 52   | 32      | 21      | 38      | 17      |
| 1C4-Spy-tag | 1            | 23   | 325  | 518  | >10,000 | >10,000 | >10,000 | >10,000 |

<1

<100

<1,000

>10,000

Supplement: S13 Fig — (A) The neutralization activities of mi3-1C4 (dark green line) and 1C4-SpyTag (light green line) against LV-based pseudoviruses of the SARS-CoV-2 D614G strain and Omicron variants. Data were collected from three technical replicates and displayed as means ± SD. The curves were analyzed by nonlinear regression (four-parameter) using GraphPad Prism (version 8.0.1). (B) A summary of the IC50 values of mi3-1C4 and 1C4-SpyTag calculated from the broad-spectrum neutralization assay in (A). (PDF) [file ppat.1013744.s013.pdf]
