# Supplementary material for: Engineering a multivalent antibody nanoparticle to overcome SARS-CoV-2 Omicron immune evasion
Source: PLoS Pathog. 2025 Dec 8;21(12):e1013744. doi: 10.1371/journal.ppat.1013744 (PMC12697983; doi:10.1371/journal.ppat.1013744)
Supplement: S1 Table — (PDF) [file ppat.1013744.s014.pdf]

| | WT-S:1C4 | BA.5-S:1C4-state 1 | BA.5-S:1C4-state 2 | BA. 5-S:1C4-interface | WT-S:8H12:1C4:3 E2 | WT-S:8H12:1C4:3 E2-interface | BA.1-S:8H12:1C4:3 E2 | BA.1-S:8H12:1C4:3 E2-interface | BA.2-S:8H12:1C4:3 E2 | BA.2-S:8H12:1C4:3 E2-interface | BA.2.75-S:8H12:1C4:3 E2 | BA.4/5-S:8H12:1C4:3 E2-state 1 | BA.4/5-S:8H12:1C4:3 E2-state 2 | XBB-S8H12:1C4:3E 2 | WT-RBD:1C4:ACE 2 |
|---|---|---|---|---|---|---|---|---|---|---|---|---|---|---|---|
| **Data Collection and processing** | | | | | | | | | | | | | | | |
| Microscope | Titan Krios G4 | Tecnai F30 | Tecnai F30 | Tecnai F30 | Tecnai F30 | Tecnai F30 | Tecnai F30 | Tecnai F30 | Tecnai F30 | Tecnai F30 | Tecnai F30 | Tecnai F30 | Tecnai F30 | Tecnai F30 | Titan Krios G4 |
| Camera | K3 | K3 | K3 | K3 | K3 | K3 | K3 | K3 | K3 | K3 | K3 | K3 | K3 | K3 | K3 |
| Voltage (kV) | 300 | 300 | 300 | 300 | 300 | 300 | 300 | 300 | 300 | 300 | 300 | 300 | 300 | 300 | 300 |
| Electron exposure dose ($e^-/Å^2$) | 48 | 60 | 60 | 60 | 60 | 60 | 60 | 60 | 60 | 60 | 60 | 60 | 60 | 60 | 48 |
| Defocus range (μm) | 1.0-1.6 | 1.0-2.2 | 1.0-2.2 | 1.0-2.2 | 1.0-2.0 | 1.0-2.0 | 1.3-2.0 | 1.3-2.0 | 0.9-1.8 | 0.9-1.8 | 1.0-2.2 | 0.9-1.9 | 1.1-2.0 | 1.0-1.6 | 0.6-1.2 |
| Pixel size (Å) | 0.650 | 0.778 | 0.778 | 0.778 | 0.778 | 0.778 | 0.778 | 0.778 | 0.778 | 0.778 | 0.778 | 0.778 | 0.778 | 0.778 | 0.650 |
| Micrographs (used) | 12,823 | 11,634 | 11,634 | 11,634 | 7,134 | 7,134 | 5,389 | 5,389 | 10,571 | 10,571 | 1,670 | 4,866 | 4,866 | 1,349 | 6,469 |
| Final particle images (nos.) | 91,916 | 148,009 | 70,759 | 148,009 | 90,363 | 90,363 | 286,938 | 286,938 | 316,199 | 316,199 | 44,969 | 25,220 | 31,904 | 13,808 | 182,640 |
| Symmetry imposed | C3 | C1 | C1 | C1 | C1 | C1 | C1 | C1 | C1 | C1 | C1 | C1 | C1 | C1 | C1 |
| Map resolution (Å) | 2.33 | 3.45 | 3.68 | 3.82 | 3.76 | 3.44 | 3.95 | 3.95 | 3.82 | 3.90 | 6.31 | 4.35 | 3.90 | 10.82 | 2.82 |
| FSC threshold | 0.143 | 0.143 | 0.143 | 0.143 | 0.143 | 0.143 | 0.143 | 0.143 | 0.143 | 0.143 | 0.143 | 0.143 | 0.143 | 0.143 | 0.143 |
| Map sharpening B factor ($Å^2$) | -48.6 | -83.9 | -72.7 | -110.2 | -70 | -93.3 | -132.2 | -120.2 | -111.3 | -129.5 | -302.8 | -51.6 | -43.4 | / | -85.9 |
| **Validation** | | | | | | | | | | | | | | | |
| MolProbity score | 1.69 | / | / | 1.95 | / | 1.58 | / | 1.81 | / | 1.73 | / | / | / | / | 1.88 |
| Poor rotamers (%) | 0.00 | / | / | 0.00 | / | 0.40 | / | 0.27 | / | 0.54 | / | / | / | / | 0.15 |
| Clashscore | 6.92 | / | / | 7.97 | / | 3.23 | / | 5.53 | / | 4.5 | / | / | / | / | 8.91 |
| RMS (bonds) | 0.0029 | / | / | 0.0044 | / | 0.0052 | / | 0.0067 | / | 0.0057 | / | / | / | / | 0.0066 |
| RMS (angles) | 0.54 | / | / | 0.72 | / | 1.11 | / | 1.16 | / | 1.19 | / | / | / | / | 0.66 |
| Ramachandran plot | | | | | | | | | | | | | | | |
| Favored (%) | 95.54 | / | / | 91.22 | / | 92.64 | / | 91.24 | / | 91.59 | / | / | / | / | 93.90 |
| Allowed (%) | 4.46 | / | / | 8.54 | / | 7.36 | / | 8.76 | / | 8.29 | / | / | / | / | 6.10 |
| Disallowed (%) | 0.00 | / | / | 0.24 | / | 0.00 | / | 0.00 | / | 0.00 | / | / | / | / | 0.00 |
