# Supplementary material for: Engineering a multivalent antibody nanoparticle to overcome SARS-CoV-2 Omicron immune evasion
Source: PLoS Pathog. 2025 Dec 8;21(12):e1013744. doi: 10.1371/journal.ppat.1013744 (PMC12697983; doi:10.1371/journal.ppat.1013744)
Supplement: Attachment — Submitted filename: Response_PlosPathogens-revisions-final.docx [file ppat.1013744.s016.docx]

**Response to Editor Comments on the Manuscript PPATHOGENS-D-25-00661.**

We thank the reviewers for recognizing the merit of our work and for their constructive comments to improve our manuscript. As you will see in the following itemized responses, we have addressed all the concerns fully and revised our paper accordingly. To facilitate the navigation of this document, we have copied the reviewers’ comments verbatim in **blue** and typed our responses in **black**.

**Reviewer #1**

*Summary:*

1. Sun et al. investigated the reduced efficacy of the broadly neutralizing antibody 1C4 against SARS-CoV-2 Omicron variants. They clarified the antibody’s recognition mechanism and the molecular basis for its diminished neutralization activity against highly immune-evasive variants such as BA.5 and XBB. By utilizing the mi3-Spycatcher nanoparticle platform to achieve multivalent polymerization of 1C4, the study significantly improved its binding avidity and successfully restored its neutralizing capacity. By integrating structural biology, cellular functional assays, and evaluations in animal models, this work proposes a viable strategy for the engineering and optimization of broadly neutralizing antibodies. This work is meaningful and offers valuable insights into addressing the ongoing evolution of SARS-CoV-2. However, the following issues still need to be addressed.

**Response:** We sincerely appreciate your positive feedback and constructive comments to improve our manuscript. We have addressed all the comments and revised the manuscript accordingly. All changes in the revised manuscript are highlighted in **red.**

*Major issues:*

1. In lines 161-168, although the N343 glycan appears to interact with 1C4 in the structural model, its direct involvement in antibody binding has not been confirmed. The authors are encouraged to perform glycan mutation or enzymatic deglycosylation experiments to determine whether the glycan is part of the epitope or indirectly contributes via conformational stabilization.

**Response:** We thank the reviewer for this insightful comment. In the original version of manuscript, we have indeed performed experiments to evaluate the role of N343 glycan in 1C4 binding (new **Fig. S5**). Specifically, we conducted enzymatic deglycosylation of the RBD by using PNGase F and observed a marked reduction in 1C4 binding, indicating the glycan contributes directly to the interaction. We have detailed analyzed these results in the revised main text (Lines 160-165, Pages 7-8, and **S5 Fig**)**.** We hope this addresses the reviewer’s concern.

2. In lines 175-180, the author analyzed the impact of the R346T mutation on antigen–antibody binding through structural modeling, but the explanation remains insufficient. In addition to the potential loss of the hydrogen bond between R346 and Y50 due to the mutation, is there a cation-π interaction between R346 and W102? If so, is this interaction disrupted by the R346T mutation? It is recommended that the author further investigate the impact of the R346 mutation in the structural analysis.

**Response:** We thank the reviewer for this valuable comment. As suggestion, we have carefully examined the local interactions between R346 and neighboring residues. In the wild-type structure, the guanidinium group of R346 is indeed positioned in proximity to the indole ring of W102, suggesting a potential cation–π interaction. Upon R346T mutation, this interaction is abolished due to the loss of the positively charged side chain, which likely contributes to reduced binding affinity. This disruption, together with the concurrent loss of the hydrogen bond between R346 and Y50, highlights the critical role of R346 in maintaining 1C4 binding. We have now included this analysis in the revised manuscript (Lines 175-176, Page 8, and revised **S4B Fig**). While beyond this study’s scope, we note that R346T emerges under immune pressure in >80% of XBB lineages (GISAID data). This mutation disrupts key interactions with 1C4 and may increases ACE2 affinity and therefore enhances infectivity, highlighting the evolutionary mechanisms of SARS-CoV-2 immune escape.

**Revised supporting information S4B Fig. The detail of cation-π interaction between R346 and W102**

3. Due to the influence of molecular flexibility, affinity estimation based on cryo-EM density intensity is not sufficiently reliable. Conclusions regarding antibody binding strength should primarily be supported by functional assays, such as surface plasmon resonance (SPR) measurements.

**Response:** We appreciate the reviewer’s suggestion regarding the use of functional assays to support conclusions on antibody binding strength. In fact, we have performed surface plasmon resonance (SPR) experiments in our original manuscript to quantitatively evaluate the binding affinity of 1C4 to different RBD variants, which provide independent support for our structural observations. The results are presented in the revised manuscript (please refer to Lines 105–112, Page 5 and **S1 Fig**).

4. The study notes that 1C4 locks WT spike in a “three down RBD” conformation, whereas the BA.5 spike remains one RBD “up”. It would strengthen the manuscript to discuss possible mechanisms for this increased RBD-up propensity in BA.5, such as trimer destabilization caused by specific mutations.

**Response:** We thank the reviewer for this thoughtful suggestion. 1C4 achieves a conformational entrapment strategy wherein antibodies stabilize the WT spike trimer in a closed, all-RBD-down configuration, thereby sterically blocking ACE2 engagement. We would like to clarify that this type of neutralization strategy has been observed in other antibodies as well, such as S2M11 (***Science.* 2020;370(6519):950-957**), 002-S21F2 (***Sci Adv.* 2022;8(40):eadd2032**), and BG10-19 (***Cell.* 2021;184(12):3205-3221.e24**). We acknowledge that It is still unclear why 1C4, as well as other reported antibodies mentioned above, showed effective “lock” capacity for some variants but not others. Although it has been suggested that trimer destabilization of the spike may be induced by specific mutations. The spike dynamics is still an exceptionally complex phenomenon that remains incompletely understood at the structural level. The neutralizing mechanisms of 1C4 against different variants is also exceptionally complex. Therefore, in the revised manuscript, we have included a discussion on the potential mechanisms underlying the increased RBD-up propensity observed in BA.5. Please refer to the revised text in Lines 485-495, Page 22.

In addition, to uncover the mechanism for 1C4 clearer, we have included additional negative-staining TEM (NS-TEM) data (revised **Fig. 6A**) showing that 1C4 binding to the WT spike not only locks the spike in the closed conformation, but also induces spike aggregation, which may also contribute to steric hindrance and therefore block ACE2 access. To better clarify this shift in neutralization mechanism, we have revised and expanded the relevant section in the Discussion part (Lines 495-499, Page 22). We believed that this revision provides a more comprehensive understanding of the neutralizing mechanism of 1C4.

5. Despite a measurable binding affinity between 1C4 and the XBB RBD, a complete loss of neutralization was observed. The underlying structural basis for this discrepancy should be further discussed.

**Response:** We have added a brief explanation in the Discussion (Lines 285-288, Page 13) to address this discrepancy. The complete loss of neutralization despite measurable binding is likely due to the R346T mutation weakening 1C4’s affinity. Since the extremely high affinity of the binding of ACE2 to variant spikes (always nanomolar levels), the residual binding of 1C4 is insufficient to block the high-affinity binding of ACE2 to the spike.

6. This study lacks a control group involving the use of mi3-Spycatcher alone to rule out potential nonspecific effects of the nanoparticle platform. It is recommended that such a control be included, or that this limitation be explicitly addressed in the Discussion section.

**Response:** We thank the reviewer for this important suggestion. We fully agree that including a nanoparticle-only control (mi3-SpyCatcher alone) would help to rule out potential nonspecific effects of the platform. However, due to the limited availability and high biosafety requirements of P3-level facilities for *in vivo* SARS-CoV-2 challenge experiments, it is difficult to include enough control groups for systematic animal evaluation at the present stage. To address this concern, we have now explicitly acknowledged this limitation in the revised Discussion section (Lines 529-541, Page 24). We appreciate the reviewer’s understanding.

*Minor Issues:*

1. In Figure 1G, the hydrogen bonding interactions involving the backbone are shown, and the participating backbone atoms should also be clearly indicated in the figure.

**Response:** Done as suggested.

2. In the Results section, detailed descriptions of experimental procedures should be minimized. It is recommended that non-essential methodological content be moved to the Materials and methods section.

**Response:** Done as suggested.

**Reviewer #2**

*Summary:*

This manuscript presents a timely and comprehensive investigation into the mechanisms of immune evasion by emerging SARS-CoV-2 Omicron subvariants and introduces an innovative antibody engineering strategy to counteract this challenge. Focusing on the broadly neutralizing antibody (bnAb) 1C4, the authors explore its reduced neutralization potency against recent variants such as BQ.1.1 and XBB—primarily due to the R346T mutation—and provide structural insights using cryo-electron microscopy. Despite a loss in neutralizing activity, 1C4 retains weakened spike binding and exhibits diminished ability to block ACE2 engagement. To overcome this, the authors employ a multivalent nanoparticle display strategy (mi3-1C4), which restores neutralization through enhanced avidity and demonstrates protective efficacy in a hamster model. This multidisciplinary approach, combining structural biology, binding assays, and in vivo studies, underscores the translational potential of multivalent antibody formats in the face of rapid viral evolution.

The study offers a strong conceptual foundation with clear relevance and scientific value. The idea that diminished antibody function can be rescued through avidity-driven engineering is both innovative and impactful. While the structural data and mechanistic insight are not yet fully integrated with the antibody engineering strategy, the study nonetheless advances the understanding of how bnAbs can be adapted to cope with emerging escape variants. The demonstration of functional rescue in both pseudovirus and live virus contexts is particularly compelling and reinforces the potential utility of this approach for therapeutic development.

That said, the manuscript requires major revisions to fully realize its potential. The presentation is currently hampered by significant language and clarity issues, as well as inconsistencies in figure labeling and data interpretation. Key methodological details are missing, and the proposed “lockdown” mechanism lacks definition and internal consistency. The inclusion of additional antibodies in the cryo-EM studies without accompanying functional data adds unnecessary complexity and may dilute mechanistic clarity. Moreover, statistical analysis is needed to strengthen the conclusions drawn from the in vivo data. The title should also be revised to more accurately reflect the scope and evidence presented in the manuscript.

In summary, this is a promising and high-impact study with clear relevance to the field of antibody therapeutics. While several aspects of execution and presentation need significant improvement, the underlying concept is strong and the data are directionally supportive of a meaningful advancement. With careful revision, improved structural interpretation, and clearer mechanistic framing, this work has the potential to make a valuable contribution to the ongoing effort to counteract SARS-CoV-2 immune escape. I encourage consideration for publication after major revision.

**Response:** We sincerely thank the reviewer for the thoughtful and constructive comments on our manuscript. We appreciate the recognition of the significance and potential impact of our study. In response to the concerns raised, we have carefully revised the manuscript to improve clarity, consistency, and completeness. All corresponding modifications have been addressed point-by-point in the following responses, and the changes have been highlighted in red in the revised manuscript.

*Major issues:*

1. The current title, “Structural-based engineering of neutralizing antibody,” is misleading, as there is no demonstrated connection between the structural findings and the antibody engineering strategy described. The manuscript does not establish how structural information guided the engineering of the mi3-1C4 construct, making the title inaccurate and the claim unsupported.

**Response:** We thank the reviewer for pointing this out. We have carefully reorganized the logical flow and revised the title to more accurately reflect the study content. The revised title reads: “Engineering a Multivalent Antibody Nanoparticle to Overcome SARS-CoV-2 Omicron Immune Evasion”.

2. The inclusion of two additional antibodies, 3E2 and 8H12, in the cryo-EM analysis is not justified. These antibodies are not evaluated in functional assays such as neutralization or animal models. If the authors intended to test synergy, it is not demonstrated. Otherwise, their presence in the structural study may confound interpretation and introduce artifacts that obscure the mechanism of 1C4 alone.

**Response:** We thank the reviewer for raising this important point, which allows us to clarify the scientific rationale for including antibodies 3E2 and 8H12 in our cryo-EM structural studies. 3E2 and 8H12 were selected because they represent structurally defined, non-overlapping epitopes with documented diverse sensitivity to Omicron mutations (***Protein Cell* 2024, 15:121-134**). Our intent in this study was not to evaluate synergy or functional efficacy of these antibodies, but to leverage their well-characterized structural and functional profiles as epitope-specific controls to address a fundamental question: How do distinct antibody epitopes differentially respond to viral evolution.

Although the three antibodies exhibited varying degrees of neutralization escape against different SARS-CoV-2 variants, our cryo-EM structures revealed that all three retained detectable binding activity to the spike proteins of every tested variant. This binding activity enabled their successful structural analyses. Critically, this collective evidence provides more compelling support than data from a single 1C4 antibody, demonstrating that epitope-binding capacity resists mutations more robustly than functional neutralization. Thus, multi-antibody cryo-EM structures provide unique insights into evolutionary constraints on antibody efficacy, informing future bispecific or multivalent designs. We have clarified this rationale in Lines 190–198, Page 9 in the revised manuscript.

3. Figure 4, which presents the animal study data, lacks any statistical analysis. Although the authors suggest that multivalent 1C4 provides enhanced protection, these claims are unsupported without statistical validation of viral load differences or protective efficacy. Including appropriate statistical tests is necessary to substantiate the conclusions drawn from these experiments.

**Response:** We thank you for highlighting this critical omission and sorry for this negligence. Incorporating robust statistical analysis is essential to strengthen the validity of the conclusions derived from our animal experiments. We have thoroughly revised **Fig. 4** and the manuscript to address this point and ensure all claims are firmly supported by statistical evidence.

*Minor issues:*

1. The manuscript contains widespread typographical and grammatical errors that interfere with readability and clarity. Examples can be found on lines 77, 125, 301, and 367, among others. A thorough proofreading and overall improvement in English usage is necessary before the manuscript can be properly evaluated.

**Response:** We sincerely thank the reviewer for pointing this out. We acknowledge that the initial submission contained typographical and grammatical errors that may have affected clarity. In response, we have thoroughly proofread and revised the manuscript to improve the overall quality of English expression. The revised version has been carefully edited for grammar, readability, and consistency throughout. We hope the improvements now meet the journal’s standards and facilitate a clearer understanding of our findings.

2. Figure legends are inadequately labeled and in some cases contain direct errors. For instance, lines 171–173 mention residues “K440” or “K44” in figure 1G, which are not visible or labeled in the figure. Additionally, in figure 2A, it is unclear which variant the antigen is derived from—this is critical information that should be clearly stated. Such omissions make it difficult to interpret the data and reduce the reliability of the figures.

**Response:** We thank the reviewer for pointing out these important issues. We apologize for the oversight and labeling errors in the manuscript. We have carefully revised the relevant description in the revised manuscript and clearly indicate the antigenic variant in **Fig. 2A**. All corresponding modifications have been incorporated and highlighted in red font in the revised manuscript.

3. Potential immunogenicity issue of using NP-based therapy is not discussed.

**Response:** We thank the reviewer for raising this important point. We have carefully reviewed the relevant literature and thoughtfully considered the potential immunogenicity concerns associated with nanoparticle-based therapies. A corresponding discussion has been added to the revised manuscript (Discussion section, Lines 529-532, Page 24) and highlighted in red font.

4. Structural modeling described in figure 1I and discussed in lines 175–183 lacks any methodological description. The manuscript should clearly state what modeling tools or approaches were used, how the models were generated, and how they were validated. This is essential for reproducibility and to assess the reliability of the conclusions drawn from these models.

**Response:** We have now added a brief description of the structural modeling approach to the Methods section (please refer to Lines 675-682, Page 31).

5. In figure S1, the binding kinetics of XBB-RBD display a sharp Kon curve and an unusually high association rate. The authors do not provide any explanation for this atypical kinetic profile. Given the importance of binding affinity in the study’s conclusions, this observation needs to be addressed and interpreted.

**Response:** As noted, the XBB-RBD exhibits a fast-on/fast-off kinetic profile in our SPR analysis. Specifically, the sensorgrams in both the association and dissociation phases lack pronounced curvature; instead, the signal rises rapidly and reaches a plateau immediately after each analyte injection. Under these conditions, the binding affinity of 1C4 to XBB-RBD is substantially reduced. Similar kinetic behaviors have been observed in previous studies, such as Liu et al. (***Science*, 2021; 372(6541):525–530**), where antibodies with low binding affinity displayed rapid association and dissociation rates. We believe that the atypical kinetics of XBB-RBD are a hallmark of reduced affinity due to viral evolvability rather than experimental artifact.

6. The cryo-EM structure of the 1C4-spike complex was already published by the authors in reference 20. The manuscript does not explain what new information the current structures provide, or why repeating the structural analysis was necessary. This lack of justification weakens the rationale for including these new structural datasets.

**Response:** We thank the reviewer for pointing out this observation. In our previous study, the reported structures involved WT spike bound by 1C4 in combination with either 8H12 or 3E2, forming two-antibody cocktails primarily aimed at demonstrating their cooperative neutralization effects, and we did analysis the 1C4 epitope and interacting interface in detail. In contrast, the current study focuses on the structure of 1C4 in complex with the spike protein alone, enabling a detailed analysis of its individual binding mode, epitope characteristics, and how recent mutations—such as R346T—affect its interaction with the spike.

7. The manuscript provides epitope-level detail for antibody binding, but does not adequately describe or analyze the paratope, i.e., the regions of 1C4 that engage the spike. Understanding the paratope is crucial for dissecting binding specificity and for any claims related to antibody engineering or escape mutations, and this should be addressed more thoroughly.

**Response:** As suggestion, we have re-analyzed the cryo-EM structure and generated a new figure that highlights the paratope of 1C4, including the CDR regions that engage the spike RBD. The updated figure (**revised S4A Fig**, also attached below) provides a clear visualization of the interacting CDR loops and their spatial relationship to the RBD epitope. We have also added corresponding descriptions to the Results (Lines 164-166, Page xx).

**Revised supporting information S4A Fig. The detail of paratope of 1C4.** (Left) Surface representation of the 1C4 Fab, with interacting residues highlighted as transparent sticks. The corresponding residues on the RBD that form the epitope are shown as purple stick. (Right) Surface hydrophobicity map of the 1C4 paratope

8. The cryo-EM structures in figure 2 are not discussed in sufficient detail. Their inclusion seems to serve only as a visual confirmation that the antibodies bind to the spike, without offering meaningful structural insight or analysis. If they are not contributing to the mechanistic understanding, the necessity of including them should be reconsidered or their interpretation expanded.

**Response:** We thank the reviewer for this helpful comment. Please also refer to our response to your Comment 2 above. We agree that structural data should contribute more than visual confirmation of binding. In the revised manuscript, we have expanded the description (Lines 233-236, Page 11) to clarify that the cryo-EM structures in **Fig. 2** provide important insights into the disconnect between binding and neutralization observed in certain variants. Specifically, although neutralization by some antibodies is completely lost, the structures show that low-level binding of antibody to the spike is still preserved. This structural evidence supports the idea that partial epitope engagement remains possible and therefore provides a prerequisite for further functional rescue through avidity-based engineering. Thus, these structures serve as a foundation for the design and optimization of multivalent or enhanced antibody formats.

9. The manuscript proposes a “lockdown” mechanism of neutralization by 1C4, but this concept is inconsistently presented. It is unclear why the mechanism would be effective for some variants but not others, especially given that all tested variants are neutralized. The authors need to clearly define what the lockdown mechanism entails and reconcile it with their binding and neutralization data.

**Response:** We sincerely appreciate the reviewer's insightful critique regarding the presentation of the "lockdown" mechanism. The term "lockdown" describes a conformational entrapment strategy wherein antibodies stabilize the spike trimer in a closed, all-RBD-down configuration, thereby sterically blocking ACE2 engagement. We would like to clarify that this type of neutralization strategy has been observed in other antibodies as well, such as S2M11 (***Science.* 2020;370(6519):950-957**), 002-S21F2 (***Sci Adv.* 2022;8(40):eadd2032**), and BG10-19 (***Cell.* 2021;184(12):3205-3221.e24**). All these antibodies are reported capable of binding to the spike protein with all RBDs locked in the “three-down” (closed) conformation. Based on these studies, the “lockdown” mechanism requires high-affinity property of antibodies to bind to RBDs in the "down" state. The mechanism’s efficacy varies across variants due to mutation-induced affinity decreasing and perturbations in spike dynamics.

We acknowledge that It is still unclear why 1C4, as well as other reported antibodies mentioned above, showed effective “lock” capacity for some variants but not others. The spike dynamics is an exceptionally complex phenomenon that remains incompletely understood at the structural level. The neutralizing mechanisms of 1C4 against different variants is also exceptionally complex. To uncover the mechanism for 1C4 clearer, we have included additional negative-staining TEM (NS-TEM) data (new **Fig. 6A and B**) showing that 1C4 binding to the WT spike not only locks the spike in the closed conformation, but also induces spike aggregation, which may also contributes to steric hindrance and blocks ACE2 access. In contrast, for the BA.2 and later Omicron variants, 1C4 fails to lock the spike in the closed conformation and even does not induce spike-antibody aggregation. Therefore, the neutralization of Omicron subvariants by 1C4 likely involves alternative mechanisms, such as inhibition of membrane fusion or prevention of S1 shedding—mechanisms that have also been proposed for antibodies like SP1-77 (***Sci Immunol.* 2022;7(76):eadd5446**). To better clarify this shift in neutralization mechanism, we have revised and expanded the relevant section in the Discussion part (Lines 485-498, Page 22). These changes have been highlighted in red font in the revised manuscript.

**Reviewer #3**

*Summary:*

In this manuscript, Sun et al. report the engineering of a neutralizing antibody to overcome immune evasion by Omicron variants. Through neutralization, binding assays and cryoEM analysis, the authors identify the R346T in recent variants as a key residue to 1C4 escape. Additionally, the RBD-up, -down confirmations observed in different variants appear to contribute to immune evasion. Displaying 1C4 on a nanoparticle promotes efficient spike aggregation and viral cross-linking through multivalent binding, thereby enhancing neutralization activity and providing protection against Beta and XBB challenge in a hamster model.
Overall, the paper is well-constructed, the study aim is clear, and the results are of interest to the field. However, some methods, figure legends lack sufficient detail, and some results do not fully support the conclusions (see specific comments below). In my view, this manuscript can be published after these issues are addressed.

**Response:** We thank the reviewer for the positive evaluation of our study and for the constructive suggestions. We have carefully addressed the concerns raised, and detailed point-by-point responses are provided below. Corresponding revisions have been made in the manuscript and highlighted in red.

*Major issues:*

1. Fig 1A and 1B, Since the BA.4 and BA.5 spike sequences are identical, it is unnecessary to show both. It would be helpful to include more detailed information about the amino acid substitutions in the spike proteins in the “Materials and methods” section. Additionally, to help orient readers to the significance of R346T, the authors should note that the only difference between BQ.1 and BQ.1.1, or BA.2.75 and BA.2.75.2 (this data would be valuable to include but is currently mission), is the R346T substitution. XBB contains both the R346T and V445P substitutions, which may collectively contribute to the reduced binding/neutralization by 1C4.

**Response:** We sincerely appreciate the reviewer’s insightful critique regarding the presentation of BA.4/BA.5 spike data and the mechanistic role of R346T. BA.4 and BA.5 indeed share identical RBD protein sequences, so, as suggested, we have now showed only the data of BA.5 in the revised **Fig. 1A** and **1B** to avoid unnecessary repetition. We have also included a summary of the RBD mutations in all of the tested spike proteins as a supplementary figure (**revised S6 Fig, also see details below**) to better illustrate the differences among variants, including the unique R346T and V445P substitutions.

**Revised supporting information S6 Fig. RBD sequence alignment of SARS-CoV-2 variants.** The residues involved in 1C4-footprint highlighted in green.

2. Figure 3A: The authors should first demonstrate the binding of the three mAbs (preferably Fabs) to the variant spike proteins using the same method, prior to evaluating their ability to inhibit ACE2 binding. If the mAb and ACE2 are mixed before being added to the spike-coated plate, their binding will largely depend on their relative affinities. It is expected that mAbs with weak binding affinity for the spike will not effectively compete with ACE2. Given that the binding footprint of 1C4 only marginally overlaps with the ACE2 binding site, it is plausible that 1C4 IgG could partially interfere with ACE2 binding through steric hindrance.

**Response:** We thank the reviewer for this valuable suggestion. In accordance with the recommendation, we have performed additional ELISA assays to evaluate the binding of the three mAbs to variant spike proteins using a consistent method. The results demonstrated that mAbs with weak binding affinity for the spike are indeed could not effectively compete with ACE2. These results have been incorporated into the revised manuscript (revised **Fig. 3A**, also attached below), and the corresponding analysis have also been added (Lines 241-242, Page 11).

**Revised Fig. 3. The binding efficacy (A) and ACE2-competitive potency (B) of antibodies with diverse spike proteins.**

As for the ACE2 binding interfere potency of 1C4, we have added an additional negative-staining TEM (NS-TEM) data showing that 1C4 binding to the WT spike could induce spike aggregation, which may also contribute to steric hindrance and blocks ACE2 access (new **Fig. 6A and B**).

3. Since several platforms aimed at enhancing antibody avidity and thereby increasing binding and/or neutralization have already been published, the authors should discuss the advantages and disadvantages and potential applications of their engineered antibody in this context.

**Response:** We appreciate the comment. As suggested, a comparative discussion of our antibody engineering approach with existing avidity-enhancing platforms has been included in the revised Discussion section (Lines 533-537, Page 24) to address potential advantages, limitations, and therapeutic implications.

*Minor issues:*

1. Fig2C, it is not clearly indicated which variant- BA.2, BA.5, or XBB-the 3 mAbs are escaping from. Based on the text (line 218), it appears that all 3 mAbs escape from XBB. This should be clarified in the figure or legend.

**Response:** We have clarified this point in the figure legend accordingly.

2. Fig 2E, There are labeling errors in the figure. 1C4-VL is incorrectly labeled as 8H12-VH, and 3E2-VH and 3E2-VL are mislabeled as 8H12-VH and 8H12-VL, respectively. Please correct these to avoid confusion.

**Response:** Sorry for this typo. We have corrected it as suggestion.

3. Figure 3F–H: In the legend, the phrase “Comparison of the ACE2 binding potency for the RBD in WT- and BA.5-spike complexed with 1C4” is misleading. The term “potency” does not accurately describe what Figures 3F–H are showing.

**Response:** We have revised the wording in the figure legend and substituted “potency” with “ability” to enhance accuracy.

4. Fig 4C, An excess amount of 1C4-spytag should be used to ensure saturation of the mi3 binding site. The authors should clarify that the mi3-1C4 used in Fig 4F and in the animal studies are the same as that shown in Fig 4C, meaning that not all binding sites may be occupied by 1C4. Discussion of potential lot-to-lot variation would also be helpful.

**Response:** As shown in the SEC purification profile attached below (**Appendix Fig. 1**), mi3-SpyCatcher was incubated with an excess amount of 1C4-SpyTag during the assembly process to ensure saturation of the available binding sites. The unbound 1C4-SpyTag was subsequently removed by size-exclusion chromatography. The same batch of purified mi3-1C4 was used consistently in both **Fig. 4F** and the animal studies.

**Appendix Fig. 1. Size-exclusion chromatography (SEC) profile for the purification of mi3-1C4**

5. Fig 5E, 5G, the y-axis label “viral RNA at copies/ml” is confusing and should be changed to copies/gram of tissues, and the full names of the organs should be used in the figures and/or legends for clarity.

**Response:** Tissues were homogenized in cold PBS at a fixed mass/volume ratio, so Viral RNA copies quantified in the homogenate (copies/ml) were directly comparable to copies per gram of tissue. We preferred to use "copies/ml" to maintain consistency with liquid sample viral load reporting. We have added a detailed homogenization protocol (Lines 709-715, Page 32) in Materials and Methods for clarify.

6. Line 377: The phrase “significantly higher” appears to be incorrect in context. Based on the values provided, the mi3-1C4-treated hamsters showed lower viral RNA levels than the untreated group. Please revise accordingly.

**Response:** Corrected as suggested.

7. Fig 6F, Please clearly describe how the samples were transferred from BSL-3 to BSL-2 in Fig 6F, specifically noting that the samples were fixed prior to transfer.

**Response:** We thank the reviewer for the reminder. The detailed procedure regarding sample transfer is described in the Materials and Methods section. To improve clarity, we have also added a corresponding note in the figure legend.

8. Line 655, “For the solid organ samples, we collect 1mg turbinate, 0.1 mg trachea and 0.1 mg lung in 1mL PBS”, did you mean grams instead of mg?

**Response:** Sorry for the mistakes, we have corrected “mg” to “g”.

**Reviewer #4**

*Summary:*

 This paper examines why antibodies lose neutralizing potency against SARS-CoV-2 variants, and how to improve upon them. They solve many cryoEM structures of neutralizing antibodies bound to spike proteins from various variants, and show that one antibody in particular (1C4) may lose ability to neutralize XBB variants because it can no longer “lock” the RBDs in the down confirmation. They go on to multimerize IC4 using a spy tag system, which increases the neutralization against XBB and beta variants in mice and has significantly better potency in vitro. They then investigate the mechanism of action of this multimerized form of 1C4 and suggest that it may be different from un-multimerized IgG. While 1C4 presented as an IgG “locks” the RBD down, multimerized 1C4 may instead cause protein/virus aggregation, thus preventing infection. This is supported by negative stain EM images that show that the multimerized antibody causes aggregation of spikes and CryoEM images of SARS-Cov02 virion that show they may aggregate together more in the presence of multimerized antibodies.

**Response:** We thank the reviewer for the insightful summary and constructive comments. We have expanded the structural analysis and mechanistic discussion of the multimerized 1C4 in the revised manuscript, highlighting its role in viral aggregation and neutralization. Please refer to the detailed responses and highlighted revisions in the updated manuscript.

*Major issues:*

1. One of the major claims in this manuscript is that multimerizing antibodies may decrease the frequency of which they need to be replaced in the clinic and “keep pace with viral evolution” but the data shown here indicates that strategy may only be practical for a small subset of antibodies. The group demonstrates the likely method of neutralization of 1C4 is “locking” of the RBD in the down conformation, and that 1C4 can still bind modern variants, but cannot lock the RBD. Surely, for antibodies that completely lose affinity to spike, multimerizing would have very little effect. This, combined with the fact that the increase (over regular IgG) in protection shown here conveyed by multimerizing 1C4 are marginal, severely diminished my enthusiasm for this manuscript. Further, it is extremely well established as a physical law of chemistry that multimerizing a protein will increase its apparent affinity (avidity effects).

There is some interesting information about the antibody 1C4 losing the ability to “lock” the RBDs in the down state over time. There is a wealth of structural data reported here, but the molecular/evolutionary mechanisms for how SARS-CoV-2 evades locking are not presented.

This may be because the resolution for the 1C4 WT spike data set is very low, based off very few particles with very few images taken, and there is no model built. Maybe this is the reason that no RBDs in the up conformation were observed? Perhaps a larger data set would reveal more classes with RBDs in the up conformation? Given that a large portion of the paper is based on the observation that 1C4 can lock the RBD, I would expect this data set to be much higher quality and have a model built to understand the molecular mechanisms. I would at a minimum expect multiple lines of evidence to be presented that support this method of neutralization.

**Response:** We sincerely appreciate the reviewer’s thoughtful and scientifically grounded critique, which raises crucial points regarding the applicability of antibody multimerization and the mechanistic interpretation of our data. Below we address each concern with additional experimental evidence and clarification:

(1) **Applicability of Multimerization Beyond 1C4:** We agree that the success of antibody multimerization strategies can be dependent on the specific epitope targeted and the intrinsic binding properties of individual antibodies. In some cases, neutralization may not be fully restored through multimerization, as revealed in previously reported ferritin-based “Multabody” platform (***Nat Commun.* 2021;12(1):3661**). Despite these limitations, these multimerized antibody strategies still hold considerable potential. To demonstrate broader utility, we extended our strategy to two additional antibodies (8H12 and 3E2, reported in our previous study ***Protein Cell*. 2024;15(2):121-134**) targeting non-overlapping epitopes, As shown in **Appendix Fig. 2 and 3 below,** multimerized mi3-8H12 and mi3-3E2 exhibited: >100-fold increase in neutralization breadth against Omicron variants XBB.1 and EG.5.1. Synergistic effects when combined with mi3-1C4 in a tri-specific nanoparticle. This confirms multimerization can rescue neutralization for diverse antibody classes, provided residual binding affinity exists.

**Appendix Fig. 2. Broad neutralization activity of mi3-8H12, mi3-3E2 and tri-specific mi3-nanoparticle**

**Appendix Fig. 3. Summary of broad neutralization activity of mi3-8H12, mi3-3E2 and tri-specific mi3-nanoparticle (Fold change of mi3-based antibodies IC₅₀ for variants relative to 614G)**

**(2) Mechanism of 1C4 Neutralization Loss in Omicron Variants:** The reviewer correctly notes that 1C4 loses "locking" capacity against newer variants. Our cryo-EM structures (revised **Fig. 3F-H**) reveal that the bind modes of 1C4 to different variants are different. 1C4 achieves a conformational entrapment strategy wherein antibodies stabilize the WT spike trimer in a closed, all-RBD-down configuration, thereby sterically blocking ACE2 engagement. We would like to clarify that this type of neutralization strategy has been observed in other antibodies as well, such as S2M11 (***Science.* 2020;370(6519):950-957**), 002-S21F2 (***Sci Adv.* 2022;8(40):eadd2032**), and BG10-19 (***Cell.* 2021;184(12):3205-3221.e24**). We acknowledge that it is still unclear why 1C4, as well as other reported antibodies mentioned above, showed effective “lock” capacity for some variants but not others. The spike dynamics is still an exceptionally complex phenomenon that remains incompletely understood at the structural level. The neutralizing mechanisms of 1C4 against different variants is also exceptionally complex. To this end, we have performed an additional negative-staining TEM (NS-TEM) analysis, which clearly shows that 1C4 induces spike aggregation even without full locking (new **Fig. S10** and **Fig. 6D**), corroborating aggregation-mediated neutralization. Although 1C4 loses its ability to lock “down” RBDs or induces spike aggregation when binding to spike proteins of later Omicron variants, residual binding enables multimerized 1C4 to induce viral aggregation (NS-TEM in **Fig. 6**), providing an alternative neutralization mechanism. Therefore, in the revised manuscript, we have included a discussion on the potential mechanisms underlying the increased RBD-up propensity observed in BA.5. Please refer to the revised text in Lines 485–498, Page 22.

**(3) Incremental Benefit of Multimerization**: We agree that for antibodies with complete affinity loss may not benefit from multimerization. However, clinically relevant antibodies often retain partial binding: 1C4’s KD for XBB is 564 nM, typical of many "evaded" therapeutics. Multimerization increased 1C4’s apparent affinity for XBB by >30,000-fold (KD 564 nM → 16.8 pM, **Fig. 4K**), translating to restored in vivo efficacy (**Fig. 5C,G**), indicating that avidity effect is non-linear. Furthermore, we would like to clarify that the apparently limited protective gain achieved by 1C4 multimerization compared to conventional IgG is likely attributable to the low pathogenicity of the virus in hamster, which may obscure the efficacy of multimeric antibodies. To address this, we further conducted histopathological analysis of animal tissues. The previous results demonstrated that mi3-1C4 reduced lung viral loads by 2-log vs. 1C4 IgG (p<0.0001, **Fig. 5G**) and prevented severe lung pathology (we provided new H&E data in **Fig. 5I**). Taken together, we believed that this approach offers a practical and translationally relevant strategy to extend the utility of otherwise weakened antibodies, without the constant need to generate entirely new clones.

**(4) Enhanced Structural Validation**: To address concerns about the WT-S:1C4 structure, we obtained additional cryo-EM data (12,823 movies) and processed of WT-S:1C4 with a larger number of 98,621 particles and yielded a 3.20-Å resolution interface map (local refinement, **S11 Fig)** , which showed all three RBDs are locked in “down” states. We built atomic model and confirmed that 1C4 stabilizes closed states preferentially. Even with high-resolution structures, it remains challenging to analyze from a structural perspective why 1C4 can lock certain parts of the WT spike in a "closed" conformation where all three RBDs are "down." As we mentioned above, the dynamics of the spike protein is an exceptionally complex issue, and the neutralization mechanism of 1C4 also exhibits multifaceted characteristics. Therefore, we have provided a more comprehensive explanation and discussion of this aspect in the discussion section. Together, these findings strengthen the proposed neutralization model, and the revised manuscript has been updated with the corresponding figures and expanded discussion.

*Minor issues:*

1.Line 135- typo

**Response:** Corrected.

2. Line 205- I do not think we can infer anything about affinity from cryoEM data sets. Affinity should refer directly to biophysical experiments that measure affinity. FAB dentistry in cryoEM can result from many factors and does not necessarily relate to affinity.

**Response:** We thank the reviewer for this important clarification. We agree that Fab density observed in cryo-EM reconstructions can be influenced by multiple factors—including flexibility, occupancy, or conformational heterogeneity—and does not directly reflect binding affinity. Accordingly, we have revised the relevant sentence in the manuscript to remove any implication of inferred affinity and instead describe the observed Fab density in more neutral terms.

3. Line 216- I am not sure that “diverse amounts of Fabs on different spikes is really relevant to the mechanism of action of these ABs. For example, differences in spike could cause slightly different FAB orientations which affects how many FABs can bind at the same time.

**Response:** We agree that the number of bound Fabs per spike may not directly reflect the mechanism of action. Our aim was just to show that the spike can accommodate all three Fabs simultaneously without interference, indicating non-overlapping epitopes. Importantly, these three antibodies represent distinct classes, and structural comparison within the same complex allows us to assess their relative binding capacity. Combined with existing affinity data, this provides molecular-level insight into their ability to engage Omicron variants.

4. Figure 2a- does not really seem to be explained in the text or figures. It is unclear to me why 3E2 was injected one separately from the other in the competition assay3.

**Response:** We thank the reviewer for pointing this out and apologize for the lack of clarity in the figure explanation. In **Fig. 2A**, 3E2 was injected separately as a control to demonstrate that it binds independently to the antigen and does not compete with the other two antibodies. We have updated the figure legends to provide a more detailed explanation of this experimental design.

5. Line 234- typo.

**Response:** Corrected.

6. Line 264- no evidence is given for why 1C4 locks some spikes but not others in the down state. Since there is a wealth of structural data presented, a structural mechanism for this should be delineated.

**Response:** We thank the reviewer for this insightful question. At present, the mechanism by which antibodies stabilize the locked (all RBD-down) conformation of the spike remains unclear. Although we observe the final conformational state in the cryo-EM reconstructions, it is difficult to accurately infer the dynamic process or triggering mechanism that leads to this outcome based solely on static structural data. We have added a brief note in the Discussion (Lines 485-498, Page 22) to acknowledge this limitation.

7. Line 456- “been proved to render the cross linking of trimeric spikes” does not quite make sense.

8. Line 458- is capable to block should be “is capable of blocking”.

9. Line 464-5 are very hard to understand what is being conveyed.

**Response:** We appreciate the reviewer’s detailed comments. In response, the original sentences corresponding to comments 7–9 have all been replaced in the revised manuscript to enhance clarity, accuracy, and readability.

10. Line 467- should be “…of virus evasion”.

**Response:** Done as suggestion.
