# Supplementary material for: Engineering a multivalent antibody nanoparticle to overcome SARS-CoV-2 Omicron immune evasion
Source: PLoS Pathog. 2025 Dec 8;21(12):e1013744. doi: 10.1371/journal.ppat.1013744 (PMC12697983; doi:10.1371/journal.ppat.1013744)
Supplement: Attachment — Submitted filename: Response_PlosPathogens-2ndrevisions-final.docx [file ppat.1013744.s017.docx]

**Response to Editor Comments on the Manuscript PPATHOGENS-D-25-00661-R1.**

We thank the reviewers for recognizing the merit of our work and for their constructive comments to improve our manuscript. As you will see in the following itemized responses, we have addressed all the concerns fully and revised our paper accordingly. To facilitate the navigation of this document, we have copied the reviewers’ comments verbatim in **blue** and typed our responses in **black**.

**Reviewer #2:**

**Summary:**

The authors have addressed most of the previous concerns; however, several issues remain unresolved or insufficiently addressed. Below are the major and minor points that still require attention.

**Response:** We sincerely thank the reviewers for their constructive comments. We have addressed the previous concerns and have revised the manuscript accordingly. All remaining points have been carefully considered, clarified, and incorporated where appropriate in the updated text.

**Major Comments:**

**Comment 1:** Addressed.

**Response:** Thanks for your feedback.

**Comment 2:** In the rebuttal, the authors explain that the inclusion of two additional antibodies in the structural analysis was intended to “address a fundamental question: How do distinct antibody epitopes differentially respond to viral evolution.” This is an important and insightful rationale. However, this motivation and its implications are not reflected in the revised manuscript. The Results and Discussion sections should explicitly elaborate on how the presented structures contribute to answering this fundamental question. Please integrate a clear discussion linking the structural data to this overarching objective.

**Response:** We thank the reviewer for raising this important point. In response, we have updated the Discussion to clarify the rationale for including 3E2 and 8H12 in the structural analysis. Specifically, we added the following text in the revised manuscript: “The inclusion of 3E2 and 8H12 alongside 1C4 allowed us to examine how distinct antibody epitopes respond differently to viral evolution. Structural analyses revealed a common feature: all three antibodies show markedly reduced binding against Omicron subvariants, yet residual interactions remain detectable, indicating that viral escape does not necessarily eliminate binding. The key difference lies in their epitope locations, which determine each antibody’s degree of sensitivity and resilience to mutations. Notably, 1C4 retains measurable binding to all tested spikes, including XBB, but its affinity is substantially weakened, preventing stable engagement of the RBD and abolishing neutralization. These findings highlight that loss of neutralization does not always equate to loss of binding.” (**Lines 508-516, Page 23**)

**Comment 3:** Addressed.

**Response:** Thanks for your feedback.

**Minor Comments**

**Comment 1:**

**a.** The authors stated that the manuscript was “carefully” and “thoroughly proofread,” yet most errors remain unchanged from the initial version, despite being specifically highlighted in the previous review.

Examples of uncorrected errors:

Line 122 (previously line 125): “…may does not lead to…”

Line 316 (previously line 301): “spacater”

In addition, numerous new errors were introduced during the revision process:

Lines 166–168: Ten residues are listed, but only nine are highlighted as the 1C4 epitope (green) in Figure S6. Residue T345 is missing.

**Response:** We thank the reviewer for carefully pointing out these issues. We have now corrected and modified all the typographical errors and missing label.

**b.** Lines 168–179: The revision removed K440, but it is now unclear which of the remaining residues mediate the salt bridge interaction.

Lines 170–173: These lines describe the wild-type sequence but cite Figure 1G, which, according to the legend, corresponds to BA.5 RBD.

Lines 171–173: The text describes N440K and cites Figure 1G, yet neither K440 nor N440 is shown or labeled in the figure.

These issues indicate that the manuscript requires another round of careful proofreading and verification to ensure consistency between text and figures.

**Response:** We sincerely thank the reviewer for the careful and patient examination. Our original intent was to clarify the role of residue 440 in the 1C4–spike interaction. Specifically, although N440K represents a recurrent mutation, our structural analysis shows that this residue is positioned at the periphery of the 1C4 epitope (Fig. 1F) and only makes ordinary contacts rather than forming critical hydrogen bonds or salt-bridges. To avoid confusion, we have revised the text to indicate that Fig. 1F illustrates the peripheral location of residue 440, while Fig. 1G depicts the hydrogen-bond and salt-bridge network, in which residue 440 is not involved. We have carefully edited the relevant sentences to ensure consistency between the text and figures (**Lines 172-175, Page 8**).

**Comment 2:** The authors claim this point has been addressed, but no revision is evident.

**Response:** We thank the reviewer for the comment. The revisions addressing this point are detailed under Comment 1-b.

**Comment 3:** Addressed.

**Response:** Thanks for your feedback.

**Comment 4:** Addressed.

**Response:** Thanks for your feedback.

**Comment 5:** The authors cite Liu et al., Science 2021, 372(6541):525–530 to justify their observation of an atypical kinetic profile. However, there are several issues: The cited paper does not include any author with the first or last name “Liu.” The cited paper used a bi-valent analyte (see Figure 1B, top two curves) and applied a 1:2 curve fitting model (Table S1). If the current study also used a bi-valent analyte, this must be explicitly stated, with clarification whether a 1:1 or 1:2 fitting model was applied in this manuscript. The cited paper reports a maximum kon of 10^5 and koff of 10^-2, whereas this manuscript reports values of 10^8 and 10^2, respectively. Such a large discrepancy strongly suggests either an experimental artifact or an unusual biological finding. This issue needs to be discussed explicitly in the text.

**Response:** We appreciate the reviewer’s valuable comments. We have carefully re-evaluated the kinetic fitting parameters and experimental settings. Upon re-examination, we found that the original values might have been overestimated due to an inappropriate fitting model. Therefore, we repeated the binding assays and reanalyzed the data. The updated SPR results are shown below (**Appendix Fig. 1**). We noticed that the fitted curves on the right panel did not align well with the running curves, as the “fast association and fast dissociation” pattern of this assay is not suitable for kinetic fitting. Instead, the binding affinity should be determined using the steady-state affinity model, which is appropriate for non–time-dependent molecular interactions. In this approach, the equilibrium dissociation constant (KD) for a 1:1 interaction is derived from the relationship between the steady-state response and the analyte concentration. Consequently, no corresponding Koff or Kon values are generated in this fitting mode. The corresponding results and updated sensorgrams have been incorporated into the revised manuscript (**Lines 111, Page 5,** and see revised **Fig. S1**).

**Appendix Fig. 1 The binding affinity of 1C4 to the RBD of spike protein of SARS-CoV-2 XBB variant.** The colored curves represent the experimental SPR sensorgrams, and the curves on the right present the local fitting results that were used to calculate the KD values according to a 1:1 binding model. The dashed lines denote the fitted curves.

**Comment 6:** Addressed.

**Response:** Thanks for your feedback.

**Comment 7:** Addressed.

**Response:** Thanks for your feedback.

**Comment 8:** The revised manuscript now justifies including the cryo-EM structure in Figure 2, noting in lines 233–236: “These structural findings… quantitatively reveal that many Omicron subvariants retain low-level antibody engagement.” However, it remains unclear how this “quantitative” assessment was conducted. Please provide a clear explanation of the methodology used to determine this quantitative measurement, or include supporting data to substantiate the claim.

**Response:** We thank the reviewer for this important comment. We realize that our original wording “quantitatively reveal” may have been misleading, as it could be interpreted as a numerical measurement of antibody engagement. Our intention was to convey that the cryo-EM structures provide molecular-level evidence of the extent of antibody binding, as reflected by the number of Fabs observed on the spike trimers across different variants. To avoid misunderstanding, we have revised the sentence to: “They provide molecular-level evidence that many Omicron subvariants retain low-level antibody engagement, as reflected by the reduced but still detectable number of Fabs bound per spike.” (**Lines 236-239, Page 11)**

**Comment 9:** In the rebuttal letter, the authors state: “Neutralization of Omicron subvariants by 1C4 likely involves alternative mechanisms, such as inhibition of membrane fusion or prevention of S1 shedding.” If the authors believe that 1C4 neutralizes different variants through distinct mechanisms, this hypothesis should be explicitly stated in the manuscript itself. The Discussion should be revised to incorporate this point, rather than leaving it only in the rebuttal letter.

**Response:** We thank the reviewer for this comment. In response, we have revised the Discussion to explicitly present the hypothesis that 1C4 may neutralize different Omicron subvariants through distinct mechanisms. We have clarified this point with the following text: “In contrast, for the BA.2 and later Omicron variants, 1C4 fails to lock the spike in the closed conformation and even does not induce spike-antibody aggregation. Therefore, the neutralization of Omicron subvariants by 1C4 likely involves alternative mechanisms, such as inhibition of membrane fusion or prevention of S1 shedding, which that have also been proposed for antibodies like SP1-77.” (**Lines 500-504, Page 22**)

**Reviewer #4:** The authors have sufficiently addressed all my concerns, and I have no further comments.

**Response:** Thanks for your feedback.
